# Supplementary material for: Can we enhance neurorehabilitation through regional implementation of group-based telerehabilitation? A mixed-methods evaluation of NeuroRehabilitation OnLine (NROL)
Source: BMJ Open. 2026 Feb 2;16(2):e101820. doi: 10.1136/bmjopen-2025-101820 (PMC12878247; doi:10.1136/bmjopen-2025-101820)
Supplement: online supplemental file 1 [file bmjopen-16-2-s001.pdf]

Supplementary file 1: Service data tables

Includes Supplementary Tables:

- Table S1: NROL therapy staff and regional community neurorehabilitation therapy workforce characteristics
- Table S2: Proportion of patients referred to NROL from wider service by organisation
- Table S3. NROL delivery and utilisation summary over time
- Table S4: NROL group delivery and attendance over 12 blocks regional NROL
- Table S5: Therapy provision for stroke patients: NROL participants vs. Non-NROL patients
- Table S6: NROL Travel avoidance
- Table S7: NROL patient characteristics over time
- Table S8: Selected characteristics of stroke patients: NROL participants vs. Non-NROL patients
- Table S9: NROL patient outcomes

Table S1: NROL therapy staff and regional community neurorehabilitation therapy workforce characteristics

	Evaluation period<br>Regional<br><br>Apr 22–Mar 24[a]	Apr 23-Mar 24[b]
**Characteristics**	**NROL**<br>**Regional therapy staff (%)**<br>**(N=75)**	**Wider community neurorehabilitation services**<br>**Regional therapy workforce (%)**<br>**(N=135)**
**Organisation**		
ELHT	41%	40%
UHMB	29%	16%
BTH	19%	23%
LSCFT	11%	21%
**Role**		
Occupational therapist	35%	27%
Physiotherapist	33%	27%
Psychology	4%	1%
Speech and Language Therapist	12%	15%
Dietician	1%	1%
Therapy assistants/trainees	15%	28%

Regional = multi-service. [a]2-year evaluation period (Regional Total). [b]Annual period

Table S2: Proportion of patients referred to NROL from wider service by organisation

	Evaluation period<br>Regional<br>Apr 22–Mar 24[a]
	**% patients referred to NROL relative to patients referred to the wider community neurorehabilitation service**
**Organisation**	
ELHT	7%
UHMB	16%
BTH	2%
LSCFT	2%

Table S3: NROL delivery and utilisation summary over time

	Annual Single-service Apr 21–Apr 22	Annual Regional Apr 22–Mar 23	Apr 23–Mar 24	**Evaluation period Regional Apr 22–Mar 24**
**Service metric**	Single-service (S)[a]	Regional 1 (R1)[a]	Regional 2 (R2)[a]	**Regional Total[b]**
**Total: Targeted therapy and community groups, entry session**				
Blocks delivered, n	6	6	6	12
Sessions delivered, n	198	255	256	511
Patients referred, n	121	192	249	438 (ELHT 46%, UHMB 27%, BTH 16%, LSCFT 11%)
Patients participated, n	98	155	187	339 (ELHT 47%, UHMB 25%, BTH 16%, LSCFT 11%)
Patient contacts, n	1057	1618	1816	3434 (ELHT 45%, UHMB 28%, BTH 16%, LSCFT 11%)
Median patients participating per block, n (range)	21 (17-26)	32 (27 -36)	38 (30 – 50)	34 (27-50)
Median sessions per participating patient, n (range)	8 (1-35)	10 (1-47)	8 (1-50)	9 (1–50)
Patient session attendance rate, %	58%	58%	55%	56%
Therapy staff contacts, n	361	585	582	1167 (ELHT 53%, UHMB 29%, BTH 10%, LSCFT 7%)
Therapy staff:patient ratio, median (range)	0.4 (0.1–3.0)	0.4 (0.1–2.0)	0.3 (0.1–2.0)	0.4 (0.1–2.0)
**Targeted therapy groups: Talking and Physical**				
Groups delivered per block, median (range)	5 (4–6)	7 (6–7)	7 (5–8)	7 (5–8)
Sessions delivered, n	163	214	214	428
Patients participated, n	96	153	184	334
Patient contacts, n	739	1091	1252	2343
Median patients per group, n (range)	4 (1-11)	5 (1-12)	6 (1-13)	5 (1-13)
Median block capacity[c], %	73%	80%	85%	84%
Patient session attendance rate, %	67%	69%	72%	71% (ELHT 65%, UHMB 73%, BTH 78%, LSCFT 83%)
Therapy staff contacts, n	293	496	466	962
Therapy staff:patient ratio, median (range)	0.5 (0.1–3.0)	0.5 (0.1–2.0)	0.4 (0.1–2.0)	0.4 (0.1–2.0)
**Community: Peer-support group[d,e]**				
Sessions delivered, n	29	35	36	71
Patients participated, n	64 (65%)	97 (63%)	104 (56%)	200 (59%)
Patient contacts, n	256	417	437	854
Median patients per group, n (range)	9 (4-14)	12.5 (6-21)	12.5 (6-20)	12 (6-21)
Patient session attendance rate, %[e]	41%	38%	32%	35%
Therapy staff contacts, n	54	72	99	171
Therapy staff:patient ratio, median (range)	0.2 (0.1–0.4)	0.2 (0.1–0.4)	0.2 (0.1–0.5)	0.2 (0.1–0.5)

Regional = multi-service. [a]Annual periods (S, R1, R2). [b]2-year evaluation period (Regional Total). Three patients participated in both annual reporting periods (R1 and R2) and are included in each. [c]% Block capacity reflects proportion of patient allocation opportunities filled at start of NROL block. [d]NROL entry sessions are only reflected in total. [e]Peer-support group is optional attendance for all patients.

Table S4: Group delivery and attendance over 12 blocks regional NROL

	**Evaluation period Regional Apr 22-Mar24[a]**							
**Group/s**	**Sessions delivered, n**	**Patients participated, n**	**Proportion patients in group, %**	**Patient contacts**	**Median group capacity, % (target group size)**	**Patient session attendance rate, %**	**Therapy staff contacts**	**Therapy Staff:patient ratio, median (range)**
**Total (incl. Peer-support group)**	511	339	100%	3434		56%	1167	0.4 (0.1 – 2.0)
**Total (excl. Peer-support group)**	440	339	100%	2580		71%	996	0.4 (0.1 – 2.0)
**Total Targeted therapy**	428	334	99%	2343		71%	962	0.4 (0.1 – 2.0)
**Talking**	287	250	74%	1426		71%	619	0.5 (0.1 – 2.0)
Cognitive education	72	64	19%	293	92% (6)	74%	163	0.6 (0.3 – 1.5)
Cognitive processing	35	38	11%	124	83% (6)	69%	78	0.7 (0.3 – 2.0)
Adjustment/wellbeing	32	79	23%	183	58% (12)	71%	68	0.4 (0.2 – 1.0)
Fatigue	83	136	40%	608	100% (12)	67%	173	0.3 (0.1 – 0.8)
Dysarthria	39	27	8%	119	67% (6)	81%	85	0.8 (0.3 – 2.0)
Aphasia	25	23	7%	96	83% (6)	83%	50	0.5 (0.2 – 1.0)
Vocation	1	3	1%	3	83% (6)	60%	2	0.7 (0.7 – 0.7)
**Physical**	141	138	41%	917		70%	343	0.4 (0.2 – 1.5)
Balance & Mobility	72	97	29%	535	88% (12)	72%	201	0.4 (0.2 – 0.8)
Upper Limb	69	80	24%	382	71% (12)	67%	142	0.4 (0.2 – 1.5)
**Total Community**	83	286	84%	1091		40%	205	0.2 (0.1 – 0.5)
NROL entry session	12	236	70%	237		81%	34	0.2 (0.1 – 0.2)
Peer-support[b]	71	200	59%	854		35%	171	0.2 (0.1 – 0.5)

Regional = multi-service. [a]2-year evaluation period (Regional Total). [b]Peer-support group is optional to attend with all patients invited to all sessions.

Therapy provision

Data information and processing:

- Stroke clinical audit datasets (for Sentinel Stroke National Audit Programme[1]) acquired from two organisations (ELHT, BTH)
- All available stroke patient records within the evaluation period were included, except patients who didn't access community services.

Table S5: Small-scale review therapy provision for stroke patients: NROL participants vs. Non-NROL patients

		**Evaluation period Regional Apr22-Mar24**										
		**NROL**					**Non-NROL**					**p-value[a]**
		n	Mean	Median	SD	IQR	n	Mean	Median	SD	IQR	
Total	Total duration of stay in service (days)	76	140.22	146.00	38.34	117-167.5	1,520	71.02	59.00	53.24	28-109	**p<0.001**
	Total therapy received (minutes)	76	2017.34	1535.00	1371.94	1015-2855	1,520	694.77	335.00	902.63	165-832.5	**p<0.001**
OT	Duration in service in OT rehabilitation (days)	68	122.51	133.50	48.04	87.5-157.5	1,086	65.02	53.00	50.63	24-97	**p<0.001**
	Total OT received (minutes)	68	753.74	755.50	556.46	300-995	1,086	288.34	175.00	342.92	90-335	**p<0.001**
PT	Duration in service in PT rehabilitation (days)	69	111.49	121.00	57.06	65-156	1,106	69.24	61.00	52.62	22-108	**p<0.001**
	Total PT received (minutes)	69	1097.99	845.00	996.54	320-1625	1,106	507.15	257.50	639.45	120-615	**p<0.001**
SLT	Duration in service in SLT rehabilitation (days)	29	94.28	90.00	64.46	32-146	508	63.16	48.00	54.04	19-95	**0.011**
	Total SLT received (minutes)	29	505.97	80.00	885.22	45-525	508	303.54	95.00	534.85	45-280	0.712
PSY	Duration in service in psychological support rehabilitation (days)	31	102.23	107.00	64.56	41-154	131	94.64	82.00	63.77	42-155	0.507
	Total psychological support received (minutes)	31	375.16	150.00	373.94	60-680	131	212.25	120.00	227.63	60-300	0.082

Stroke patient data only. Fours care domains = Occupational therapy (OT), Physiotherapy (PT), Speech and Language Therapy (SLT) and Psychological Support (PSY). [a]Mann-Whitney U test. NROL = NROL participants. Non-NROL patients = not referred to NROL or referred but did not start.

NROL Travel avoidance

Data information and processing:

- Informed estimates of avoided driving miles and time were determined using Google maps, based on referring staff worksite and patient residential postcode combined with NROL session attendance
- Mileage cost avoidance 59 pence per mile (NHS mileage rate[2])
- Avoided carbon emissions estimated using total avoided mileage cost and a UK conversion factor (0.517 kgCO^2e per £[3])

Table S6: NROL Travel avoidance

	Annual Regional Apr 22–Mar 23	Apr 23–Mar 24	**Evaluation period Regional Apr 22–Mar 24**
**Travel metric**	Regional 1 (R1)[a]	Regional 2 (R2)[a]	**Regional Total[b]**
Avoided driving miles, n	38364	37191	75554
Avoided driving time, hours	1254	1252	2506
Avoided mileage cost, £	22635	21942	44577
Avoided carbon emissions, kgCO_2e	11702	11344	23046

Regional = multi-service. [a]Annual periods (R1, R2). [b]2-year evaluation period (Regional Total).

Table S7. NROL patient characteristics over time

Characteristics	Annual Single-service	Annual Regional		Evaluation period Regional		
	Apr 21–Apr 22	Apr 22–Mar 23	Apr 23–Mar 24	**Apr 22–Mar 24**		
	Single-service (S)[a] Participants (N=98)	Regional 1 (R1)[a] Participants (N=155)	Regional 2 (R2)[a] Participants (N=187)	**Regional Total[b] Participants (n=339)**	**Regional Total[b] Did not start (n=99)**	**P-value[c]**
Organisation						
ELHT	98 (100%)	80 (52%)	82 (44%)	161 (47%)	40 (40%)	
UHMB		46 (30%)	41 (22%)	86 (25%)	33 (33%)	
BTH		16 (10%)	38 (20%)	54 (16%)	14 (14%)	
LSCFT		13 (8%)	26 (14%)	38 (11%)	12 (12%)	
Age years, median (range)	57 (19–86)	60 (20–89)	60 (19–89)	60 (19–89)	63 (22-89)	**0.023**
Gender, male (%)	58 (59%)	90 (58%)	100 (54%)	190 (56%)	50 (51%)	0.302
Ethnicity						
White	73 (75%)	140 (90%)	164 (88%)	301 (89%)	91 (92%)	
Asian British or Asian	10 (10%)	8 (5%)	13 (7%)	21 (6%)	5 (5%)	
Black British or Black	-	2 (1%)	2 (1%)	4 (1%)	-	
Other	-	3 (2%)	2 (1%)	5 (2%)	-	
Not reported	15 (15%)	2 (1%)	6 (3%)	8 (2%)	3 (3%)	
Index multiple deprivation, median (range)	4 (1–10)	5.5 (1–10)	5 (1–10)	5 (1–10)	5 (1 – 10)	0.670
Rurality, rural (%)	18 (18%)	42 (27%)	38 (20%)	79 (23%)	17 (17%)	0.196
Living alone, yes (%)	14 (14%)	26 (17%)	31 (17%)	57 (17%)	25 (26%)	0.053
Condition						**0.009**
Sudden	73 (75%)	137 (88%)	175 (94%)	310 (91%)	98 (99%)	
Stroke	45 (46%)	109 (70%)	150 (80%)	257 (76%)	85 (86%)	
Progressive/intermittent	25 (26%)	18 (12%)	12 (6%)	29 (9%)	1 (1%)	
Chronicity						
Subacute (1 week - <6m)	46 (47%)	89 (57%)	138 (74%)	225 (66%)	79 (80%)	**0.011**

Single = single-service, Regional = multi-service. [a]Annual periods (S, R1, R2). [b]2-year evaluation period (Regional Total). Three patients participated in both annual reporting periods (R1 and R2) and are included in each. [c]Mann-Whitney U or Chi-square. Participants with missing values for a given variable were excluded from that specific analysis only; missingness was minimal (all <2%).

Table S8. Small-scale review of selected characteristics for stroke patients: NROL participants vs. Non-NROL patients

Data information and processing:

- Stroke clinical audit datasets (for Sentinel Stroke National Audit Programme[1]), acquired from two organisations (ELHT, BTH)
- All available stroke patient records within the evaluation period were included, except patients who didn't access community services.

	**Evaluation period Regional Apr22-Mar24**										
	**NROL**					**Non-NROL**					
**Characteristic**	**n**	**Mean**	**Median**	**Min**	**Max**	**n**	**Mean**	**Median**	**Min**	**Max**	**p-value[b]**
Age, years	76	61.03	60.50	31	89	1520	72.71	75.00	18	100	**<0.001**
Gender, % male[a]	76	57.89	-	0	1	1520	56.58	-	0	1	0.821
Pre-stroke disability, mRS	56	0.41	0.00	0	4	1267	0.87	0.00	0	5	**<0.001**
Admission stroke severity, NIHSS	56	6.80	4.00	0	28	1267	6.21	4.00	0	42	0.386
Index multiple deprivation	76	4.46	4.00	1	10	1506	4.24	4.00	1	10	0.413
Rurality, % rural[a]	76	7.89	-	0	1	1505	14.22	-	0	1	0.120

Stroke patient data only. [a]Median values not provided for binary variables. [b]Mann-Whitney U or Chi-square. NROL = NROL participants. Non-NROL = patients not referred to NROL or referred but did not start.

NROL patient outcomes

Data information and processing:

- Health-related quality of life was measured by the EQ-5D-5L consisting of two parts: the EQ descriptive system profiles individual's health state and EQ Visual Analogue Scale (EQ-VAS, max 10) rates individual's perceived overall current health.[4,5,6] Descriptive system question responses were transformed into an EQ Index score (EQ-Index, max 1.000) using the EuroQoL Group's crosswalk methodology with a United Kingdom population value set[7]
- Activity performance measured by the Patient Specific Functional Scale (PSFS, max 10), was used to generate patients' average rating on performing activities of function that are important to them and which were impacted by their neurological condition[8,9,10] (Stratford et al. 1995, Mathis et al. 2019, Evensen et al. 2020)
- Mean differences were provided for comparison with minimal clinically important differences (MCIDs), defined as EQ-Index≥0.10, EQ-VAS≥10, and PSFS≥2.8 points[6,9]

Table S9: NROL Patient outcomes

	**Evaluation period Regional Apr 22 – Mar 2024**						
**Measure**	**N**	**Median entry (IQR)**	**Median exit (IQR)**	**p-value[a]**	**Mean difference (SD)**	**% NROL participants who improved or remained stable (change score (exit-entry) ≥ 0)**	**% NROL participants met or exceeded MCID**
EQ-Index	280	0.537 (0.315-0.689)	0.605 (0.434-0.728)	**<0.001**	0.070 (0.203)	64%	39%
EQ-VAS	280	60 (50-74)	60 (50-75)	**0.009**	3.03 (19.02)	68%	36%
PSFS	282	3.33 (2.33-4.67)	5.00 (3.67-6.75)	**<0.001**	1.62 (2.03)	80%	30%

[a]Wilcoxon-Signed Rank Test. MCID = Meaningful clinically important difference

References

1. SSNAP | Sentinel Stroke National Audit Programme 2025 [Available from: https://www.strokeaudit.org/Home.aspx. (Accessed 5 February 2025)
2. NHS Employers. Mileage allowances FAQs. 2023 [Available from: https://www.nhsemployers.org/articles/mileage-allowances-faqs] (Accessed 5 February 2025).
3. Department for Environment, Food and Rural Affairs. Carbon footprint for the UK and England to 2021. 2021 [Available from: https://www.gov.uk/government/statistics/uks-carbon-footprint. (Accessed 5 February 2025)
4. Euroqol. EQ-5D-5L. [Available from: https://euroqol.org/information-and-support/euroqol-instruments/eq-5d-5l/] (Accessed 27 January 2025).
5. Golicki D, Niewada M, Buczek J, Karlińska A, Kobayashi A, Janssen M, et al. Validity of EQ-5D-5L in stroke. Quality of life research. 2015;24:845-50
6. Chen P, Lin K-C, Liing R-J, Wu C-Y, Chen C-L, Chang K-C. Validity, responsiveness, and minimal clinically important difference of EQ-5D-5L in stroke patients undergoing rehabilitation. Quality of life research. 2016;25:1585-96.
7. Van Hout B, Janssen MF, Feng YS, Kohlmann T, Busschbach J, Golicki D, Lloyd A, Scalone L, Kind P, Pickard AS. Interim scoring for the EQ-5D-5L: mapping the EQ-5D-5L to EQ-5D-3L value sets. Value in health. 2012 Jul 1;15(5):708-15.
8. Stratford P, Gill C, Westaway M, Binkley J. Assessing disability and change on individual patients: a report of a patient specific measure. Physiotherapy canada. 1995;47(4):258-63.
9. Mathis RA, Taylor JD, Odom BH, Lairamore C. Reliability and validity of the patient-specific functional scale in community-dwelling older adults. Journal of Geriatric Physical Therapy. 2019;42(3):E67-E72.
10. Evensen J, Soberg HL, Sveen U, Hestad KA, Bronken BA. The applicability of the patient-specific functional scale (PSFS) in rehabilitation for patients with acquired brain injury (ABI)–A cohort study. Journal of multidisciplinary healthcare. 2020:1121-32.
