# Supplementary material for: Can we enhance neurorehabilitation through regional implementation of group-based telerehabilitation? A mixed-methods evaluation of NeuroRehabilitation OnLine (NROL)
Source: BMJ Open. 2026 Feb 2;16(2):e101820. doi: 10.1136/bmjopen-2025-101820 (PMC12878247; doi:10.1136/bmjopen-2025-101820)
Supplement: Supplementary file 3 [file bmjopen-2025-101820.reviewer_comments.pdf]

PEER REVIEW HISTORY

BMJ Open publishes all reviews undertaken for accepted manuscripts. Reviewers are asked to complete a checklist review form and are provided with free text boxes to elaborate on their assessment. These free text comments are reproduced below.

ARTICLE DETAILS

Title (Provisional)

Can we enhance neurorehabilitation through regional implementation of group-based telerehabilitation? A mixed-methods evaluation of NeuroRehabilitation OnLine (NROL)

Authors

Ackerley, Suzanne; Mason, Thomas Daniel; Partington, Adam; Peel, Rosemary; Vernon, Helen; Connell, Louise

---

VERSION 1 - REVIEW

**Reviewer**	**1**
**Name**	**Ganesh, Shankar**
**Affiliation**	**Composite Regional Center for Persons with Disabilities, Physiotherapy**
**Date**	**13-Aug-2025**
**COI**	**None**

The manuscript may contribute to elucidating the role of tele-rehabilitation as an alternative to community rehabilitation services. Regrettably, the material is composed in such a complicated and incoherent manner that it becomes challenging to understand the work. The authors assert that the NeuroRehabilitation OnLine (NROL) constitutes a hybrid model of care, presumably encompassing both digital and in-person services; however, the description fails to specify the nature of the in-person community rehabilitation provided, including the organisation of the groups, among other details. The introduction characterises NROL as a group-based telerehabilitation program, whereas later sections describe it as supplementing existing care. This must be explicitly clarified. The 'innovation' section lacks clarity, and the authors' references to '6-week blocks' and terms like 'net-zero' and 'per café NROL session' are ambiguous. Kindly elucidate the inclusion criteria for participation in NROL, the acceptance criteria, and the methodology of therapy delivery at patients' residences. When did the patient exit from the NROL? The open-ended questions and the exemplary quotes offer minimal contribution to the study.

Another restriction is the 'biased' nature of the work, as no information has been disclosed regarding the data evaluators. The perception is that the same stakeholders conduct the

conception, implementation, and evaluation of the program, thereby compromising its transparency. The authors are to provide information pertaining to qualitative data gathering and analysis. The authors have documented a diverse range of system-level consequences on page 9. We recommend that the authors present the results in a consistent format. Considering the attendance percentage, dropout rates, and survey data, do the authors ascertain that the NROL has successfully achieved its scaling-up objectives? Please provide data on non-NROL participants who enrolled within the same timeframe for comparative analysis. Where can I find the data regarding therapy provision throughout all four domains of care? The illustration on page 31 is complex; kindly verify the percentage of individuals who have completed NROL.

**Reviewer**	**2**
**Name**	**Lakhani, Ali**
**Affiliation**	**La Trobe University, Department of Public Health**
**Date**	**22-Oct-2025**
**COI**	**None**

Introduction: The introduction is sound; however, a stronger summary of teleneurorehabilitation—particularly its impact on clients and health systems—is warranted. While the introduction notes that its use has been effective, it does not clearly describe findings related to implementation as evaluated in the manuscript. If there are no other implementation studies which exist, this is fine, yet it needs to be stated and justified as to how authors have come to this conclusion.

The research aim as described is broad (lines 20-27). There are distinct research questions which are addressed via the approach, which need to be clearly stated at the end of the introduction. For example, what were clients and staff perspectives of NROL? What factors contributed to utilisation for patients? etc.

Methodology: The mixed methods approach is robust, with adequate measures used. However details of the approach are non-existent. A distinct overarching summary of the approach, and data collection (including retrospective) from each group, needs to be provided as a signpost at the start. Figure 1 is clear however is not comprehensive. For example, no where in figure one is it stated that there would be a comparison of NROL current and non-NROL retrospective clients, on particular outcomes.

Findings: The findings are generally clearly described, however are all outcomes as detailed within the methods section presented? For example, were findings from inferential analysis of factors contributing to NROL, or comparison of non-NROL and NROL participants on outcomes presented. The authors should revise the findings section so that it clearly responds to each form of analysis proposed in the methods.

Discussion: The discussion emphasises implementation processes and considerations, yet does not speak to the patient level data considered. This may be due to word limitations.

Overall feedback: In light of previous feedback noting that not all analyses proposed in the methods are presented in the findings, and that the introduction does not clearly articulate distinct research questions, it may be worthwhile to revise the manuscript, and focus specifically on domains (populations, outcomes, measures etc) which exist throughout the entire manuscript. For example aligning the research questions, methods, findings and discussion on the distinct aims which are addressed throughout.

VERSION 1 - AUTHOR RESPONSE

	Comment	Response	Page/ section
Reviewer 1			
1	The manuscript may contribute to elucidating the role of tele- rehabilitation as an alternative to community rehabilitation services. Regrettably, the material is composed in such a complicated and incoherent manner that it becomes challenging to understand the work.	Our apologies. We have taken on both reviewers' comments and have undertaken major revisions to improve the readability of the manuscript. This includes streamlining the narrative of the paper under the selected implementation outcomes (systems-level adoption, acceptability and sustainability) and moving content to the supplementary files to allow improved focus on the main findings.	All
2	The authors assert that the NeuroRehabilitation OnLine (NROL) constitutes a hybrid model of care, presumably encompassing both	Thank you for highlighting this point. It is important that it is clear that NROL delivers its therapy remotely via an online platform only. While NROL contributes to a hybrid rehabilitation offer when embedded within an existing neurorehabilitation care pathway, NROL itself is not a hybrid model. Instead,	

	digital and in-person services; however, the description fails to specify the nature of the in-person community rehabilitation provided, including the organisation of the groups, among other details.	when adopted, it sits alongside existing therapy (e.g. home-based in-person, onsite group, and online individual therapy) broadening the overall rehabilitation offer.<br><br>To clarify this aspect, we have amended/added sentences in key places in the manuscript.	
	The introduction characterises NROL as a group-based telerehabilitation program, whereas later sections describe it as supplementing existing care. This must be explicitly clarified.	Intro: "*Embedded within the service's established neurorehabilitation care pathway, NROL contributed to the rehabilitation offer, sitting alongside existing therapy provision as part of a hybrid model of care*."<br><br>Methods/'Innovation': *"Embedded into established neurorehabilitation care pathways, the NROL programme aimed to improve access to therapy by providing telerehabilitation to complement the existing rehabilitation offer."*<br><br>*"Clinical reasoning determined if and when an NROL programme fitted within a patient's overall rehabilitation."*<br><br>With the above clarified, we felt that the current description of the nature of the existing rehabilitation offer within the 'Inner setting' section was suitable with minor edits. However, we shifted the 'Inner setting' section to precede the 'Innovation' section to improve flow (i.e. providing an explanation of existing	Pg 5 'Introduction'<br><br>Pg 7 'Innovation'<br><br>Pg 7 'Innovation'

		rehabilitation offer before describing how NROL complements this).<br><br>Methods/'Inner setting': *"Before NROL's scale-up (April 2022), services existing rehabilitation offer primarily consisted of in-person individual evidence-based therapy at patients' home, with limited onsite group and occasional online*<br><br>*individual therapy sessions. The exception was ELHT, who have provided online*<br><br>*group therapy via NROL at a single-service level since January 2021."*	Pg 7<br><br>'Inner setting'
3	The 'innovation' section lacks clarity.	To improve clarity, we have added further detail to the section including the programme aim and more information about the types of groups (e.g. cognitive, communication, well-being, mobility, upper-limb, peer-support) and<br><br>their frequency (i.e. weekly for six-weeks). A paper describing NROL as a regional innovation in more detail (using the TIDieR checklist) remains signposted.	Pg 7, 'Innovation'

4	The authors' references to '6-week blocks' and terms like 'net-zero' and 'per café NROL session' are ambiguous.	We have addressed these points as follows:<br>- More detail has been added to the innovation section to help understand the concept of the 6-week 'NROL block'<br>- 'Net-Zero' has been replaced with 'environmental targets'<br>- 'Café NROL' has been replaced by 'Peer-support group'	Throughout

5	Kindly elucidate the inclusion criteria for participation in NROL, the acceptance criteria, and the methodology of therapy delivery at patients' residences. When did the patient exit from the NROL?	The inclusion/acceptance criteria for participation in NROL is situated within 'Methods/'Individuals'' and reads as shown below. However, we have added a sentence to clarify that the patient remained under the responsibility of the local treating therapist. If you feel this summary requires any further details, please let us know.<br><br>Methods/Individuals: "*In addition to service eligibility, inclusion required English proficiency (or translator access) and willingness to participate in online group therapy, with or without carer support. Participants required access to suitable computer hardware or connectivity, though support was sought from Age UK. Patients remained part of a local treating therapist's caseload who was ultimately responsible for their overall rehabilitation*"<br><br>We believe the query regarding 'methodology of therapy delivery at patients' residences' has been addressed by response 2.<br><br>To clarify patient exit from the NROL programme we have now included the following sentence:<br><br>Methods/ 'Innovation': "*Patients typically exited the programme at the end of their 6-week block, however could attend more than one NROL block if clinically indicated providing they remain under the active care of their stroke or neurorehabilitation team*."	Pg 7/8 'Individuals'<br><br>Pg 7 'Innovation'

6	The open-ended questions and the exemplary quotes offer minimal contribution to the study.	We appreciate your opinion. For the service evaluation we considered it important to capture both quantitative and qualitative perceptions from key stakeholders to inform the implementation outcomes (system-level: adoption, acceptability, sustainability). Inclusion of open-ended questions allowed deeper insight into the challenges and enablers of implementation, the perceived value of NROL, and key considerations for sustained use. Themes derived from CFIR-guided framework analysis of the open-ended questions were merged with quantitative survey findings to inform summaries under CFIR-headings of the system-level constructs influencing implementation (Table 3). Notably, some summaries only arose from qualitative enquiry.<br><br>We have revised the manuscript to make the contribution of the qualitative aspect of the survey more explicit.<br><br>Methods/Data collection and analysis:<br>*"The surveys used a mix of multiple choice, Likert-scale rating, and open-ended questions. The open-ended questions were used to provide deeper insights into implementation challenges and enablers, perceptions of NROL's value, and key considerations for its sustained use."*<br><br>Text in the discussion remains: "*A strength of a mixed-methods approach is that the use of qualitative and quantitative data can help illustrate converging or*	Pg 9<br>'Data collection and analysis'<br><br>Pg 25<br>'Discussion'

		diverging factors, providing a more comprehensive view."	
7	Another restriction is the 'biased' nature of the work, as no information has been disclosed regarding the data evaluators. The perception is that the same stakeholders conduct the conception, implementation, and evaluation of the program, thereby compromising its transparency.	Detail of the evaluators is situated in the 'Evaluator characteristics and reflexivity' section of the methods. However, we have now added to this section to acknowledge the potential bias our embedded roles may have introduced and our mitigation strategies.<br>Method/Evaluator characteristics and reflexivity:<br>"*This project was part of a clinical academic partnership, with LC & SA being both experienced researchers and physiotherapists in neurorehabilitation who have been involved in NROL development, implementation, evaluation, and sustainment. AP is a physiotherapist who works as the operational manager of NROL for Lancashire and South Cumbria. RP is an occupational therapist at UHMB who delivers NROL. HV is a speech and language therapist and Head of Service for Stroke and Neurorehabilitation at ELHT and provides strategic leadership for NROL. TM is an applied econometrician working as a quantitative researcher focused on health and health care*. *The evaluators' embedded role in NROL implementation provided valuable contextual insight. However, they remained mindful of potential bias and took steps to mitigate this. Evaluation was guided by logic models and informed by regular engagement with diverse stakeholders. Findings and interpretations were discussed and agreed with the wider stakeholder group involved in NROL.*"	Pg 13<br>'Evaluator characteristics and reflexivity'

8	The authors are to provide<br><br>information pertaining to qualitative data gathering and analysis.	Qualitative data was gained from the free-text responses to the open-ended questions within the staff and patient surveys. In the original manuscript, the details of data collection and analysis were embedded within the 'Survey data' section.<br><br>In the process of revising the methods section we have taken the opportunity to review the level of detail pertaining to the qualitative data and have provided some further detail within the methods and more clearly outlined the components of the survey data in a new Table (Table 1).	Pg 9<br><br>'Data collection and analysis'<br><br>Pg 11/12<br><br>'Table 1'
9	The authors have documented a diverse range of system-level consequences on page 9. We<br><br>recommend that the authors present the results in a consistent format.	Thank you for this recommendation, which is consistent with feedback from Reviewer 2. The manuscript has been revised to provide a more consistent format throughout and addresses this point.<br><br>The introduction now includes three distinct research questions outlining how implementation success was conceptualised and explored relative to the<br><br>selected implementation outcomes 'System-level: Adoption, Acceptability and Sustainability'. These questions were used to reframe the format of the<br><br>methods and results sections, enabling a more streamlined narrative.<br><br>The methods section now clearly describes the 'system-level indicators' relevant to each implementation outcome. Further detail regarding the<br><br>system-level indicators is now predominantly reported in the new Table 1. We<br><br>believe this table provides an easier format to demonstrate data alignment	'Introduction'<br>'Methods'<br><br>'Results'<br><br>Pg 11/12<br><br>'Table 1'

		with the implementation outcomes and system-level indicators. It also details indicator questions, their operationalisation and analysis approach.	
10	Considering the attendance percentage, dropout rates, and survey data, do the authors ascertain that the NROL has successfully achieved its scaling-up objectives?	Yes, we do. In the original manuscript, the concept of implementation success was discussed within the first paragraph of the discussion and depicted in the joint display (Fig 4). However, in revising the manuscript we have made improvements that we believe makes this perspective easier to discern.<br><br>We have now more clearly defined our conceptualisation of implementation success by including three research questions in the introduction. In addition, we have revised the opening paragraph of the discussion to respond more directly to the study objective and these questions.<br><br>Discussion:<br>*"This observational mixed methods evaluation of the system-level scale-up of NROL, a group-based telerehabilitation innovation, demonstrated enhancement of our regional community neurorehabilitation services. Despite anticipated healthcare system pressures, NROL was widely adopted and successfully embedded within real-world practice at all intended organisations across the region, with growing participation supported by collaborative system-level working. Acceptability was evidenced by the programme being well-utilised and valued by patients and therapy staff, while safely supporting*	Pg 5 'Introduction'<br><br>Pg 23 'Discussion' Paragraph 1

		increased therapy provision. Successful adoption, acceptability, and model efficiencies indicate strong potential for sustained delivery, although continued stakeholder engagement and alignment remain essential for long-term sustainability."	
11	Please provide data on non-NROL participants who enrolled within the same timeframe for comparative analysis.	This service evaluation used real-world routinely collected data, with access to data on non-NROL patients limited. Some comparative analysis with non-NROL patients was enabled through services' stroke clinical audit data (within the same timeframe i.e. Apr22-Mar24), but community datasets were only available from two organisations. Audit data for other neurological conditions was unavailable for all organisations.	
		The stroke audit data acquired allowed small-scale comparative review between patients with stroke who participated in NROL ('NROL') vs. those who did not ('Non-NROL'). Analysis was limited to the variables collected for the audit. Relevant comparisons were undertaken for therapy provision (amount of therapy, duration in service) and across selected patient characteristic groups (age, gender, pre-stroke disability (mRS), admission stroke severity (NIHSS), deprivation and rurality).<br><br>In the original manuscript, we presented results relating to these comparisons within the 'Therapy provision' and 'Participation' sections. However, we	Pg 11/12<br>'Table 1'

		acknowledge the non-significant findings and full discipline specific results were not presented.<br><br>In the revised manuscript, the comparative analyses undertaken have been more clearly outlined in Table 1, with a summary included in the main-text (see below).<br><br>Methods/Data collection and analysis:<br>"Comparative analysis with inferential statistical testing was used to quantify therapy provision between patients with stroke who participated in NROL<br>('NROL') and those who did not ('Non-NROL' i.e. wider service patients not referred to NROL or referred but did not start NROL). It was also used to evaluate sustained appropriateness (representativeness of NROL participants to the patient population, NROL patient outcomes (within-subject) consistent with single-service NROL evaluation19). See Table 1 for detail."<br><br>Full tables of the comparative analyses are now included in Supplementary file 1 (Tables: S5,S8,S9)<br><br>The limitations related to comparative analysis are acknowledged in the 'Strengths and Limitations' and 'Discussion' sections of the manuscript.	Pg 9<br>'Data collection and analysis'<br><br>Supplementary file 1:Tables<br>S5,S8,S9<br>'Strengths and Limitations'<br>Pg 25,<br>'Discussion'

		In regard to patient outcome comparisons, this was not possible as the patient-reported outcomes relevant to our study (EQ-5D-5L, PSFS) were not routinely collected by the wider service during the evaluation period.<br><br>However, notably, from October 2024 the EQ-5D-5L was introduced within the national community stroke clinical audit dataset (SSNAP) and may allow<br><br>comparative and longer-term review in the future. This point has been added to the discussion as follow:<br><br><br>**Discussion:** *"Consistent with staff perceptions and previous single-service NROL evaluation[19]), quantitative results indicated positive shifts in participants activity performance and health-related quality of life, supporting the programme's sustained appropriateness when delivered at a regional level. In line with the prior findings, clinically meaningful group-level differences were not detected, although meaningful gains were reported for some individuals*<br><br>*(30-39%). This may not be surprising given the relatively short duration of the NROL programme (typically six-weeks) and mixed conditions. From October*<br><br>*2024 the EQ-5D-5L has been included within the national community stroke clinical audit (e.g. SSNAP[47]) which may enable longer-term, and comparative, stroke patient outcome review within future evaluation."*	Pg 23/24<br><br>'Discussion'
12	Where can I find the data regarding therapy provision throughout all four	Our apologies, these have now been included data relating to the four	Supplementary file 1:Table S5

	domains of care?	domains of care within a table in Supplementary file 1 (Supplementary Table: S5)	
13	The illustration on page 31 is complex; kindly verify the percentage of individuals who have completed NROL.	The number of referred patients who completed NROL has now been included within the main text of the results as follows:<br><br>Results/Adoption: "*339 patients participated (77%), with 293 (67%) of referred patients completing their programme.*"<br><br>Minor edits were made to Figure 2.	Pg 14<br>Results 'Systems-level Adoption: NROL Participation'<br><br>Figure 2

Reviewer 2			
Introduction	The introduction is sound; however, a stronger summary of teleneurorehabilitation—particularly its impact on clients and health systems—is warranted. While the introduction notes that its use has been effective, it does not clearly describe findings related to implementation as evaluated in the manuscript. If there are no other implementation studies which exist, this is fine, yet it needs to be stated and justified as to how authors have come to this conclusion.	We appreciate your advice. We have edited paragraph two of the introduction, adding further detail including on patient and health system impact in relation to implementation considerations.	Pg 4 'Introduction' Paragraph 2

Introduction	The research aim as described is broad (lines 20-27). There are distinct research questions which are addressed via the approach, which need to be clearly stated at the end of the introduction. For example, what were clients and staff perspectives of NROL? What factors contributed to utilisation for patients? etc.	We agree that the objective could be better articulated by detailing distinct research questions. Therefore, we have added three questions to the end of the introduction to signpost readers to the outcomes that will be evaluated (see below).<br><br>Introduction: *"Our objective was to evaluate the scale-up of NROL, embedded within established stroke and neurological rehabilitation pathways across multiple services at four healthcare organisations, from a system-level perspective. Implementation success was conceptualised by exploring the following questions:*<br><br>*As a regional innovation:*<br><br>*1) Was NROL adopted across the region?*<br>*2) Was NROL accepted and a way to support increased therapy provision?*<br>*3) What key considerations help inform NROL's sustainability?"*<br><br>We have used these questions to frame the reformatting of the paper for a more streamlined narrative (see following responses for detail).	Pg 5 'Introduction'

Methodology	The mixed methods approach is robust, with adequate measures used. However details of the approach are non-existent. A distinct overarching summary of the approach, and data collection (including retrospective) from each group, needs to be provided as a signpost at the start. Figure 1 is clear however is not comprehensive. For example, no where in figure one is it stated that there would be a comparison of NROL current and non- NROL retrospective clients, on particular outcomes.	We appreciate this suggestion, and have undertaken a major reformatting of the methods section to address this and your overall feedback, and comments from Reviewer 1.<br><br>The methods section now begins with "*Real-world implementation of NROL was examined through an observational exploratory service evaluation"* and provides an overview of the 'system-level indicators' relevant to each implementation outcome (e.g. *Adoption: NROL participation and delivery across organisations, and challenges and enablers to adoption* etc)."<br><br>The design section now begins with: "*A mixed-methods convergent parallel design was used to evaluate NROL scale-up and is overviewed in Figure 1.*<br>*Service data (quantitative) were collected throughout the evaluation period (April 2022-March 2024), with NROL service data analysed concurrently.*<br>*Survey data (quantitative and qualitative) were collected from service staff (June - July 2023) and NROL patients (April 2022 – August 2023) and analysed separately. Results relating to the system-level indicators were synthesised and interpreted under selected implementation outcomes*."<br>The 'Data collection and analysis' section has been significantly reformatted. A summary statement signposts readers to a new Table 1, which overviews the evaluation components and demonstrates data alignment with relevant implementation outcomes/system-level indicators and analysis approaches. Additional details for service and survey	Pg 6 - 10 'Methods'

		data are summarised in the main text, including an overview of the main data sources and the comparative analyses (see below).<br><br>Methods/Data collection and analysis:<br>"*Comparative analysis with inferential statistical testing was used to quantify therapy provision between patients with stroke who participated in NROL ('NROL') and those who did not ('Non-NROL' i.e. wider service patients not referred to NROL or referred but did not start NROL). It was also used to evaluate sustained appropriateness (representativeness of NROL participants to the patient population, NROL patient outcomes (within-subject) consistent with single-service NROL evaluation[19]). See Table 1 for detail."*<br><br>Edits have also been made to enhance the comprehensiveness of Figure 1.	

Findings	The findings are generally clearly described, however are all outcomes as detailed within the methods section presented? For example, were findings from inferential analysis of	Thank you for this recommendation. The results section has been reformatted to align with the implementation outcome and system-level indicators outlined in the revised methods. Further, we have reviewed/revised the content of the results section to ensure that all results are presented in the main text or their placement in the supplementary files signposted.<br><br>Comparison of NROL and non-NROL patient outcomes was not possible as the patient-reported outcomes relevant	'Results'<br>'Supplementary files'<br><br>Pg 23/24<br>'Discussion'

	factors contributing to NROL, or comparison of non-NROL and NROL participants on outcomes presented. The authors should revise the findings section so that it clearly responds to each form of analysis proposed in the methods.	to our study (EQ-5D-5L, PSFS) were not routinely collected by the wider service during the evaluation period. However, from October 2024 the EQ-5D-5L was introduced within the national community stroke clinical audit dataset (SSNAP dataset) and may allow comparative review in the future. We have now included as a discussion point (see Discussion point below).	
Discussion	The discussion emphasises implementation processes and considerations, yet does not speak to the patient level data considered. This may be due to word limitations.	Yes, we were cognisant of word count and implementation processes and considerations were prioritised as a system-level perspective was the primary focus of the paper. However, given both reviewers have raised points alluding to patient level data we have added a short discussion point as follows:<br><br>Discussion: “Consistent with staff perceptions and previous single-service NROL evaluation19), quantitative results indicated positive shifts in participants activity performance and health-related quality of life, supporting the programme’s sustained appropriateness when delivered at a regional level. In line with the prior findings, clinically meaningful group-level differences were not detected,	Pg 22- 25<br>‘Discussion’

		although meaningful gains were reported for some individuals (30-39%). This may not be surprising given the relatively short duration of the NROL programme (typically six-weeks) and mixed conditions. From October 2024 the EQ-5D-5L has been included within the national community stroke clinical audit (e.g. SSNAP47) which may enable longer-term, and comparative, stroke patient outcome review within future evaluation.”	
Overall feedback	In light of previous feedback noting that not all analyses proposed in the methods are presented in the findings, and that the introduction does not clearly articulate distinct research questions, it may be worthwhile to revise the manuscript, and focus specifically on domains (populations, outcomes, measures etc) which exist throughout the	Thank you for your constructive feedback. We have taken on your advice, and that from Reviewer 1, and reformatted and revised the manuscript to align with the selected implementation outcomes, including revising the supplementary files.	All

	entire manuscript. For example aligning the research questions, methods, findings and discussion on the distinct aims which are addressed throughout.		

VERSION 2 - REVIEW

Reviewer 2

Name Lakhani, Ali

Affiliation La Trobe University, Department of Public Health

Date 09-Jan-2026

COI

The revisions have satisfactorily addressed my previous comments.
